# Rethinking Food Production: Nexus of Mobile Phones and Production Cost Minimization

**DOI:** 10.3390/ijerph17072457

**Published:** 2020-04-03

**Authors:** Gershom Endelani Mwalupaso, Xu Tian, Xianhui Geng

**Affiliations:** 1 China Center for Food Security Studies, College of Economics and Management, Nanjing Agricultural University Nanjing, Nanjing 210095, China; rinscod@gmail.com (G.E.M.); xutian@njau.edu.cn (X.T.); 2 Department of Agriculture and Agribusiness, Prince G Academy and Consultancy, Kabwe 10101, Zambia; 3 School of Natural Resource, Copperbelt University, Kitwe 10101, Zambia; 4 School of Agriculture, Paglory University, Kabwe 10101, Zambia

**Keywords:** mobile phone use, cost efficiency, maize production, production cost minimization, Zambia

## Abstract

Information and communication technologies are a ready tool for all strata of society and are indeed redefining the way almost everything is done. Mobile phone technology, in particular, plays a vital role in expediting improvement in the efficiency of the household resource through access to information on various available technologies. Can mobile phones improve the cost efficiency of agricultural production? Comprehension of such effect is critical, especially in the context of the Sustainable Development Goals. We addressed this topic using cross-sectional data from smallholder maize producers in Zambia. The Stochastic Frontier Analysis was applied to estimate cost efficiency. The results indicate that mobile phone use improves the cost efficiency of maize production significantly and as such, adopters have made a rational decision to adopt mobile phone use for information access. Precisely, we found a 10.2% efficiency gap in favor of users. Nevertheless, if non-users were to adopt mobile phones for agricultural information access, maize production per hectare would increase by 21.38%. Eventually, food production would be increased in an environmentally friendly manner and the price of maize would be set at a competitive price within the region because agricultural inputs would be allocated cost efficiently. Therefore, in an attempt to minimize production cost in food production, this study strongly endorses the use of mobile phones for agricultural information access.

## 1. Introduction

Sub-Saharan African governments have made various efforts to improve agricultural practices, seed quality, production cost minimization strategies, and resource allocation among other farming pursuits. Of interest to this study is the promotion of mobile phone use (hereafter called MP use) in accessing agricultural information to expedite improved production efficiency. Currently, the mobile phone is changing the face of agriculture in Sub-Saharan Africa (SSA) due to its popularity. It has overtaken the number of fixed line telephone, thereby enhancing connectivity and increasing opportunities in agriculture [1,2,3]. In view of this fact, there is a growing appeal among development practitioners, policymakers and in scholarly circles for more scientific exploration on the development effects of mobile phones and their influence in agriculture [4,5].

With the predicted increase in income levels and a growing population, agricultural production is equally expected to expand cost effectively. Arguably, the use of mobile phone in accessing information is a modern technology in agriculture and may have broader effects [6] such as reduced production costs. The current production system in agriculture suffers from traditional farming practices consisting of a lack of information search, and the outcome has been low cost efficiency and production far below Food and Agriculture Organization (FAO) estimated potential [7]. To increase agricultural output, maintain profitability even at low producer prices and become more competitive, increased access to agricultural information is recommended as it facilitates improvement in allocation of inputs such as labor, fertilizer, land, and seeds [8]. 

Masuki et al. [9] highlighted the following key drivers in explaining the growing trend of mobile phones in agriculture especially among poor producers: (a) pervasive connectivity and low-cost, (b) more affordable tools and flexible, (c) advances in data exchange and storage, (d) innovative partnerships and business models, and (e) open access movement and social media, i.e., the democratization of information. They further added that the above drivers continually shape the prospects for effectively using mobile phones in agriculture. Aker [10] also contended that the reduction of certain transaction and search costs has further fueled the subscription of farmers to mobile phones for information collection. 

Consequently, the expansion of mobile phones has elicited numerous studies providing evidence of their benefits in agriculture, such as enabling the adoption of new practices and improving farmers’ production practices [11,12], improved marketing decisions and market efficiency [13,14,15,16], enhanced agricultural productivity [17], ameliorates food and nutrition security [18] and developed better connection in agri-food [19]. While seemingly contradictory, there is little theoretical reason to believe that access to mobile phone technology would lead to an improvement in agricultural production cost efficiency. The reason is that the literature on how increased information access can lead to production cost minimization is limited and unclear.

Interestingly, despite the subject of maize production cost being recurrent and a main focus of policy debates in Zambia, it remains poorly understood. Apart from farmers’ ability, access to information is omitted in explaining the differences in production costs within the communities in the country. Moreover, the price of maize fluctuates considerably every year due to domestic production volatility [20]. Currently there is a public outcry from consumers regarding the cost of maize which has resulted from higher production costs. The irrefutable fact is that maize provides more than half of all calories consumed as it is the most predominately consumed food in the country.

The option here is to rethink farmers’ performance regarding cost efficiency, which will eventually culminate in rethinking the agricultural policies. The landlocked country enjoys remarkable potential as a prospective regional maize exporter on account of its abundant subsurface water, a vast swath of cultivable farmland, low population density, and proximity to high deficit maize markets. Thus, the promotion of MP use in collecting agricultural information could be an essential element in increasing the comparative advantage of Zambian maize producers in the region. Moreover, efforts to ensure that production costs are minimized would provide a satisfactory return for smallholder farmers and an incentive to continue maize production, and at the same time support the government in accomplishing and harmonizing its different national policy goals [21]. Unfortunately, policy considerations of this nature have never benefitted from empirical analysis as regards to the potential production cost reduction on account of MP use adoption.

If MP use contributes to the effective functioning of agriculture by improving access to agricultural information, farmers’ production cost efficiency is consequently expected to improve. However, Tadesse and Bahiigwa [16] contend that new technology adoption by farmers may not essentially imply that farmers optimally use the technology and capitalize on the benefits of the potential of the technology. Therefore, this study addresses the effects of MP use in accessing agricultural information on production cost efficiency. The results of the study provide policymakers with appropriate information to understand how to make maize production competitive and derive potential alternative policy options to promote cost efficiency. It is cardinal to rethink farmers’ performance through further delve of production cost efficiency, and especially that such studies are location-, crop- and even time-specific. 

Our study contributes to the literature in four ways. First, this paper attempts to link mobile phone use in accessing agricultural information to production cost efficiency. This is very important because the measurement of efficiency is essential especially since it is a productivity growth factor that is highly useful in gauging the potential of farmers in meeting the rising global food demand. Secondly, to the best knowledge of the researchers, no study has explicitly and empirically dealt with the potential effect of MP use on production cost reduction in smallholder farming, a central investment focus that supports broad-based poverty alleviation and food security in Africa. Thirdly, lessons of production cost efficiency of farmers are vital especially in view of the United Nations vision 2030 which advocates for innovative and cost-effective food production. Finally, we also include predictions of potential cost reduction per hectare if non-user households adopted MP use—presenting farm level evidence of what the performance of farmers would be. This has important policy implication because lower costs of production are strongly correlated with maize becoming more competitive and profitable. 

The rest of this paper is organized as follows: Section 2 describes the data and the empirical strategy employed; Section 3 presents the empirical results, discussion and policy implication; and the last section provides the conclusion.

## 2. Material and Methods

### 2.1. Study Area and Data

The data used in this study were collected through a household survey in the Central province of Zambia—Mkushi District. The area has the highest maize producing farmers in the province and it stands out as an agrarian belt of the country owing to the existing farm blocks. The district has a population of 148,814 people as of the country’s 2010 census, and most of the farmers in the 22 agricultural camps rely on rain-fed hoe farming. The soils are relatively fertile and the climatic conditions are ideal for agriculture. Major crops grown are cassava, corn, cotton, peanuts, sorghum, sugarcane, and tobacco. Remarkably, over the past years, maize growing smallholder farmers have been steadily increasing translating into increased crop production that has facilitated a positive contribution to the country’s national food security. 

The household was the basic unit for research within each of the camps with no predefined sampling units. A questionnaire was the instrument used for data collection. Content validity, which was employed in this study, is a measure of the degree to which data collected using a particular instrument represents a specific domain or content of a particular concept. Two professors and practitioners in the field of agricultural economics thoroughly evaluated the questionnaire to ensure it was adequate and fit for the intended purpose. In addition, to achieve proper meaning and interpretation, the instrument was pre-tested among smallholder farmers in the study site, and necessary amendments were made based on feedback before conducting data collection. 

The data collection was conducted from June to November 2018. In total, 201 farm households were selected through a two-stage sampling technique. Details are contained in Mwalupaso et al. [22].

A summary of household characteristics and other variables used is shown in Table 1. Interestingly, the study area is covered by at least one mobile network operator (MNO) with reasonably good coverage. The mobile penetration rate in the area stands at 53.73 %. Non-users (MP) have larger cultivated lands, more farming experience and households belonging to cooperatives, household heads with relatively higher basic education, and are located further away from markets. On the other hand, the education of the household head’s spouse of users is significantly higher than non-users. 

### 2.2. Measurement of Key Variables

The primary explanatory variable of interest was MP use in accessing agricultural information at the household level. This was a dummy variable where 1 = user and 0 otherwise. This was also specified as our treatment variable. We considered a household as an MP user if at least one adult household member owned and used a mobile phone for agricultural information collection during the survey year.

Regarding the outcome variable, we were particularly interested in production cost efficiency derived after stochastic frontier analysis (SFA) cost function. The exact procedure undertaken to derive the cost efficiency scores of each household is presented in Section 2.3. Total production cost (C), total maize output (Y) and unit prices (W) of the inputs used (fertilizer in kilograms, land in hectares, seeds in kilograms and labor in labor days) were used to obtain the outcome variable of interest. To allow for extrapolation towards national and regional scales, general equilibrium effects on input prices were determined.

### 2.3. Econometric Framework and Estimation Strategy

The study made use of SFA and t-test. While the SFA was employed to model farmers’ cost efficiency, the t-test was applied to robustly estimate the average treatment effects of mobile phone adoption on cost efficiency on a matched sample. The literature and data available were the grounds on which explanatory variables were selected.

#### 2.3.1. Production Cost Efficiency

The stochastic frontier function has undergone considerable extension following the pioneering work of Aigner et al. [23] and Meeusen and van Den Broeck [24]. Unlike the data envelopment analysis (DEA), the SFA yields a more reliable result when there are significant measurement errors and provides a specific function form. For this reason, it has become one of the most widely employed methods to analyze efficiency and productivity [25]. Our study also adopts this model where the basic form of the cost function can be described as follows:

(1) $$C_{i}=f\left(Y_{i}\;W_{i};\beta \right)\exp\left(V_{i}+U_{i}\right),$$

where $C_{i}$ is the total expenditure incurred by the ith farmer to produce $Y_{i}$ output; $W_{i}$ is a vector of farm input prices; β is a vector of parameters to be estimated, $V_{i}$ are random errors and $U_{i}$ is the cost inefficiency.

The specification of the cost function is highly essential in productivity and efficiency analysis [26] as it implies a proper determination of parameters and statistical associations in the model [27]. The likelihood ratio (LR) test as applied in Mwalupaso, Wang, Rahman, Alavo and Tian [17] was used to select the best model between the cobb-douglas and translog cost fuction. The test reveals that the parameters of CD are appropriate and suitable as confirmed by the statistical insignificance of the LR test (Chi2 = 10.33). Therefore, we employ the CD cost model and apply the approach by Wang and Schmidt [28] of estimating the parameters in both the stochastic frontier and the inefficiency model simultaneously using the maximum likelihood estimation (MLE) method. We also follow the recommendation of Kumbhakar and Lovell [29] before estimation by making the cost frontier linearly homogeneous in input prices. We do so by dividing the total cost of production and the prices of inputs by the price of land before estimation. The CD cost frontier and its inefficiency model is specified as follows:

(2) $$\ln\left(\frac{C_{i}}{W_{li}}\right)=\beta_{0}+\beta_{y}lnY_{i}+\beta_{n}\sum_{n=1}^{3}\ln\left(\frac{W_{ni}}{W_{li}}\right)+v_{i}+u_{i},$$

(3) $$U_{i}=\beta_{0}+\beta_{1}MP\;use_{i}+\beta_{i}X_{i}+z_{i},$$

where $C_{i}$ is the total production expenditure incurred to produce $Y_{i}$ output of maize, $W_{ni}$ is a vector of the three classical input prices (fertilizer, seeds, labor) of each ith household divided by the price of land ($W_{li}$), $\beta_{0}$, $\beta_{y}$ and $\beta_{n}$ are parameters to be estimated, $u_{i}$ is a non-negative inefficiency component that follows a truncated-normal distribution, $v_{i}$ is a random error following a normal distribution and $X_{i}$ and $z_{i}$ are vectors of explanatory variables and the error term in the cost inefficiency model.

As a robust check, the above estimation was also done on a matched sample. Particularly, propensity score matching (PSM) by means of a one-one matching procedure was employed to derive observations with comparable characteristics [30]. According to Bravo-Ureta et al. [31], this helps in addressing biases stemming from observed variables.

#### 2.3.2. T-Test

Given that the adoption of MP is endogenous, a mere comparison of the cost efficiency means has no causal interpretation. Therefore, conducting a robust analysis to test the causal effect of MP use on cost efficiency is essential. To achieve the study’s objective of establishing the effect of MP use on cost efficiency, a t-test was performed. In view of the matching achieved through the use of PSM as earlier indicated, robust and consistent estimation of the treatment effect would be easily generated by means of a t-test. The difference in cost efficiency between the two groups would be attributable to the adoption of mobile phones since the characteristics between adopters and non-adopters are comparable. 

## 3. Results and Discussion

### 3.1. Determinants of Production Cost Efficiency 

The empirical results of the CD cost function are presented in this section. The model specification test result of the LR test as already established is insignificant. Overall, farmers have 44.4 percent potential to reduce the production cost of maize (Table 2). This value is a signal that urgent farmers’ proactive action is required to address the flexible production costs.

Regarding the cost inefficiency section in Table 2, MP use, farming experience of household head, family size and distance from the market are significant determinants. Consistently with our expectation and common sense, the more a farmer gains farming experience, the more likely they are to improve their cost efficiency due to adequate technical skills on procurement and management of crops. However, the increase in family size increases inefficiency, and this is attributed to labor being over utilized. Most farmers in the study site practice labor-intensive agriculture as they rely on rain-fed hoe farming. With the majority being poor, maize means food and an important source of their income. Therefore, more labor is deployed within families more than is necessary. We also found that distance from markets positively impacts cost inefficiency. This is in agreement with reality because being further away from market centers may entail higher prices of basic farming inputs. 

MP use positively impacts cost efficiency. Mobile phone users have access to useful and timely information that may improve their production cost efficiency. Informed by focus group discussions held prior to the questionnaire pretest, mobile phone users are actively involved in exchanging text messages, receiving agricultural production information from farming input suppliers, mobile money services and making and receiving phone calls. Considering that most farmers are on farmer input support programme (FISP), there are frequent calls and texts exchanged between farmers and their cooperatives, family members, input suppliers, and extension officers. Maize is a dominantly produced in Zambia and as such, farmer organizations are valuable sources of information about weather, market prices, pest prevention, cost minimization practices, nutrition, type of inputs and other social facilities. Consistently with previous studies [32,33], farm households who use mobile phones access such information faster as its spread reaches many of them within a short time frame. This could be one of the propellers of MP use in maize production. 

**Figure 1:**
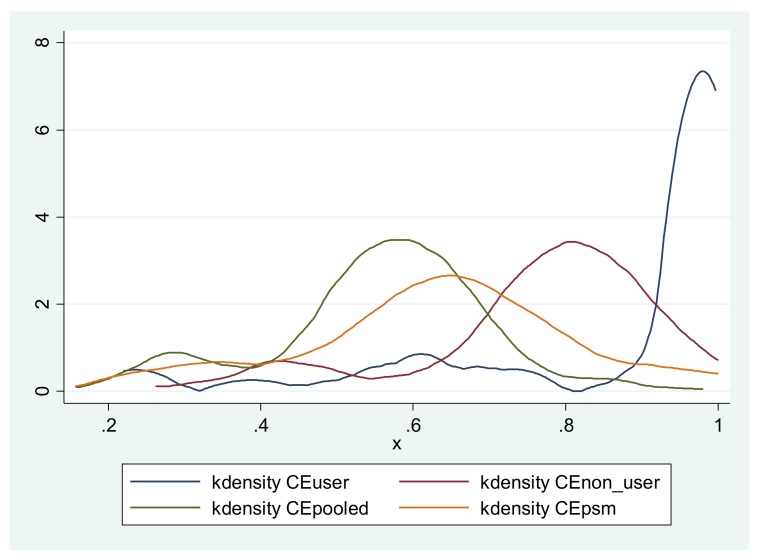
Kernel density distribution for the cost efficiency distribution. Notes: CEuser, CEnon_user, CEpooled and CEpsm represent cost efficiency distribution for users, non-users, pooled unmatched sample and for the pooled matched sample respectively.

Most importantly, when looking at the impact of such an innovation, it is vital to distinguish between presence (technology available, i.e., MP use in this case) and intensity (the frequency of accessing agricultural information) because the mobile phone is only an instrument. Table 3 presents information on the information aspect accessed, how many farmers use a particular channel to access the information, the source of the information and the frequency of information access in a month. The results intuitively disclose that farmers make the effort to (i) initiate the contact and this can be deduced by the intensity of access between farmers, the consistency in access and by mere ownership of mobile phones; (ii) discriminate which information to access as can be seen by the difference in the intensity of access per month for the different information aspects and; (iii) use that information appropriately, which is confirmed by the higher cost efficiency reported in Figure 1. However, it is essential to note that transmitting information is different from transmitting expertise in that the latter is difficult to manage asynchronously.

### 3.2. Impact of MP Use on Production Cost Efficiency

**Figure A1:**
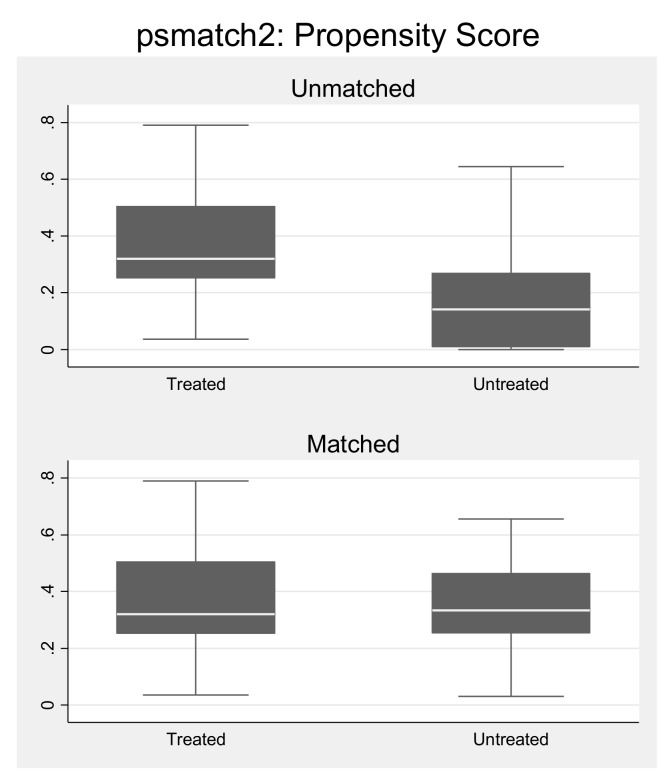
Box plot before and after matching.

Following the estimation of the cost efficiency of each respective farmer from the two groups-adopters and non-adopters, it is important to establish whether there is a casual relationship. To this end, all the characteristics between the two groups must be the same except for the treatment. Usually, this is done through matching and in this study, PSM was conducted before the SFA estimation. This implies that there are no significant differences between the two groups for all the variables under consideration (refer to Appendix A; Figure A1). Since one to one matching is achieved, each adopter is matched with a comparable non-adopter and thus, there is a balance in the observed characteristics. Therefore, the results of the t-test after matching presented in Table 4 constitute the average treatment effect. The thorough evaluation procedure establishes a significant treatment effect in a manner that a conclusion to surmise that mobile phone use positively influences cost efficiency would not be dismissed. 

The results suggest that if non-users adopted mobile phone, they would improve their cost efficiency by about 17%. This finding is peculiar to Mkushi agricultural camps and must not be generalized. Given the various means and intensity of information access revealed in Table 3, this finding is no surprise. As guided by Aker [34] and Shimamoto, et al. [35], information is a valuable resource and we contend that it makes all the difference for farmers seeking to improve their cost efficiency. Therefore, the decision to adopt is rational as it has economic benefits for farm households. Particularly, cost efficiency is significantly in favor of users at 1% significance level, which gives a percentage change of 17.82.

### 3.3. Policy Implication

According to FAO, maize is one of the staple foods of most communities in sub-Saharan Africa (SSA), and evidence indicates that over 650 million people consume about 43 kilograms of maize/person annually. In Zambia, over 50 percent of farmers allocate more than half of their cultivated area to the production of maize as it is also a major source of cash for smallholder farmers. Furthermore, the demand for maize for animal feed, industrial use, and food is growing rapidly because of the burgeoning population across the region. Undoubtedly, the role maize plays in smallholder farmers’ livelihood and food security is critical and as such lowering production cost has serious policy implications. 

Our results provide strong evidence of the positive impact of mobile phone adoption on production cost efficiency in rural Zambia. However, leveraging the full benefits of the adoption will require increased investments and policy support. Table 5 presents the policy implication calculations. If policy was directed at making all non-adopting farmers to users of mobile phones in accessing agricultural information, the 10.20% gap in cost efficiency would no longer exist between the two groups. This would translate to a 1527.64 ZMK (USD 101.50 at USD 1 = ZMK11) reduction in production cost per hectare and would eventually lead to about a 21.38% increase in maize production if the same production expenditure is incurred. This is in agreement with the United Nation’s suggestion to rethink how food is produced as it can culminate in generating decent incomes, supporting people-centered rural development and producing in an environmentally friendly manner—adequate chemicals applied during production. Moreover, the potential reduced production cost per hectare would be 19.84% less than the average production cost in southern African region (USD 700). This would make maize sold in regional markets competitive.

Therefore, the adoption of mobile phones in agriculture for collecting information is imperative to promote and sustain cost efficiency progress, and empirical evidence which is site-specific is obligatory to facilitate policy-making. However, investments in information dissemination and mobile enabled technologies must only be a prerequisite if the desired results are to be achieved. In addition, development policies aimed at agricultural transformation is also required to aggressively increase the use of and access to mobile phones in rural Zambia. Such investments will have a significant impact in improving the cost efficiency and competitiveness of maize sold.

## 4. Conclusions

To ensure that policymakers and practitioners do not think of farmers more than is warranted by their performance, this study presents strong empirical evidence on how the adoption of mobile phones in agriculture can improve production cost minimization. The reduction of production cost has many implications, such as improved profits, poverty alleviation, contribution to the fight against global hunger and increased output on account of the reduced cost of production per hectare. In view of vision 2030 regarding the SDGs, understanding such effects is of great importance. 

Traditional agriculture has undeniably been reformed by agricultural informatization advanced through the introduction of mobile phones. Overall, the findings suggest that access to information via the mobile phone improves farmers’ cost efficiency in maize production. Our results inform current policy discussion on the cost of maize production and potentially lead to the identification of unused levers capable of increasing farmers’ competitiveness. We, therefore, recommend the promotion of mobile phone use beyond regular communication in agricultural communities to guarantee an optimal production cost efficiency. To further promote the use of mobile phones in an effort to effectively improve cost efficiency, policymakers must ensure three important features: quality of information, timeliness and trustworthiness. This is pivotal because leveraging the full potential of information dissemination through the mobile telephone requires substantial enhancements in the supporting infrastructure and systems.

Finally, it is true that findings from one specific setting should not be widely generalized, but the surveyed smallholder farm households in Central Zambia are to a large extent typical of the African small-farm sector regarding access to markets and other infrastructure, mobile phone adoption and network reception, farm sizes, agricultural practices, and other social parameters. Therefore, some broader lessons can be learned. Nevertheless, follow-up investigations using panel data and a different setting will not only be useful to corroborate our findings but also extend the research direction further.

## Figures and Tables

**Table 1:** Descriptive statistics of households.

Variable	Description	Pooled Sample (201)	MP Non-User (159)	MP User (42)
Maize Price	Average price of 50kg bag of maize in Zambia kwacha (ZMK)	60.67(0.148)	60.57 (0.126)	61.07 (0.526)
Culand	Size of farm land for maize cultivation (hectares)	1.75 (0.094)	1.83 (0.110)	1.45 * (0.167)
Market	Distance to nearest output market (kilometers)	8.50 (0.261)	9.03 (0.284)	6.47 *** (0.531)
Farmexp	Farming experience of household head (years)	21.71 (0.872)	22.67 (1.01)	18.07 ** (1.53)
Family size	number of people in a household	6.14 (0.234)	6.09 (0.265)	6.31 (0.505)
Education	Household head has attended basic education (1 = yes)	0.69 (0.033)	0.81 (0.031)	0.26 *** (0.068)
Marital Status	Household head is married (1 = married)	0.77 (0.030)	0.79 (0.032)	0.69 (0.072)
Spouse Edu	Household head’s spouse attended basic education (1 = yes)	0.52 (0.035)	0.48 (0.040)	0.69 ** (0.072)
Cooperative	Household is a member of a cooperative (1 = yes)	0.93 (0.018)	0.97 (0.014)	0.79 *** (0.064)
Power Access	Households with access to power (1 = yes)	0.29 (0.032)	0.30 (0.037)	0.26 (0.069)
Gender	Gender of household head (1 = male)	0.86 (0.025)	0.86 (0.027)	0.83 (0.058)
MPownership	Owning a mobile phone (1 = owns)	0.54 (0.04)	0.42 (0.04)	0.98 *** (0.02)
MPuse rating	Perception on MP use (categorical variable)	2.18 (0.09)	1.84 (0.08)	3.5 *** (0.16)

Notes: Figures in parentheses are standard errors of the means, while *, **, and *** indicate statistical significance levels at 10%, 5%, and 1%, respectively. The significance level is determined for the difference in means between the users and non-users for each respective variable and this is indicated in the column for users.

**Table 2:** SFA Maximum Likelihood estimates.

Variables	SFA			Robust SFA
	User	Non-Users	Pooled	Pooled
Output	1.114 (0.091) ***	0.524 (0.059) ***	0.506 (0.057) ***	0.529 (0.078) ***
Fertilizer	−1.498 (1.233)	2.413 (0.458) ***	0.994 (0.485) **	0.361 (0.806)
Seed	1.781 (1.245)	0.163 (0.386)	0.475 (0.411)	0.770 (0.687)
Labor	1.755 (0.330) ***	0.557 (0.107) ***	0.596 (0.107) ***	0.766 (0.208) ***
Constant	−2.638 (4.867)	12.809 (2.278) ***	6.223 (2.277) ***	4.689 (3.273)
Inefficiency function				
MP use			−0.158 (0.066) **	−0.205 (0.080) **
Education	−0.833 (0.337) ***	−0.002 (0.006)	0.045 (0.049)	0.012 (0.015)
Farmexp	0.016 (0.010) *	−0.006 (0.003) **	0.000 (0.003)	−0.012 (0.007) *
Gender	−0.347 (0.195) *	−0.073 (0.055)	−0.096 (0.058) *	−0.013 (0.110)
SpouseEdu	−0.121 (0.211)	0.027 (0.065)	0.156 (0.057) ***	0.115 (0.084)
Cooperative	0.180 (0.133)	0.414 (0.057) ***	−0.017 (0.104)	−0.036 (0.149)
Family size	0.229 (0.037) ***	0.063 (0.009) ***	0.076 (0.011) ***	0.100 (0.015) ***
Market	0.181 (0.031) ***	0.002 (0.005)	0.011 (0.005) **	0.016 (0.010)
Culand	−0.374 (0.223) *	0.093 (0.024) ***	0.094 (0.027) ***	0.068 (0.042) *
Age	−0.111 (0.015) ***	0.057 (0.041)	−0.003 (0.003)	0.070 (0.088)
Constant	1.693 (0.297) ***	−0.634 (0.108) ***	0.298 (0.141) **	−0.309 (0.204)
Model Diagnostics				
Log-likelihood	−9.111	12.542	−13.986	−15.705
Wald chi^2^	191.04 ***	152.48 ***	128.12 ***	79.59 ***
Mean	0.867	0.764	0.556	0.629
Observations	41	159	201	82

Notes: Figures in parentheses are standard errors of the coefficient, while *, **, and *** indicate statistical significance levels at 10%, 5%, and 1%, respectively.

**Table 3:** Presence and intensity of information access via MP.

Information Aspect	Information Source	Percentage of Farmers Using			Average Frequency Per Month
		Calls	Messages	Both	
Agricultural Extension	Ministry of Agriculture	90.48			4
Prices of maize seeds	Farm Input Suppliers			100	6
Prices of fertilizer				100	6
Labor availability related to maize	Farmer Organizations			42.86	2
Cooperative meetings and communication with progressive farmers regarding maize production	Cooperatives and Farmers			100	3
Weather forecasts	MNOs		25		2
Mobile money			83.33		4

Notes: The table only considers mobile phone users.

**Table 4:** Average treatment effect of productive cost efficiency.

MP Usage Status	N	Mean	Treatment Effect	% Change
Users	41	0.680	0.102*** (0.038)	17.65
Non-users	41	0.578		

**Table 5:** Policy implication calculation.

Variable	Matched Sample
Production cost efficiency gap (average percentage change of all algorithms used)	17.65%
Current cost per hectare in ZMK	8655.18
Potential cost per hectare in ZMK	7127.54
Potential reduction cost per hectare (difference of current and potential cost) ZMK	1527.64
Actual output in kg per hectare	2988.37
Potential cost per kg (Potential cost per hectare /Actual output per hectare) ZMK	2.39
Potential increase in output per hectare (Reduced cost per hectare/potential cost per kg)	638.91
Average production cost per hectare in southern African region in ZMK	19.84%

Notes: The average cost of production in southern Africa ranges 600−700 USD. We used 700 USD at the exchange rate of 11 ZMK = 1 USD.
